# A detailed EIS study of boron doped diamond electrodes decorated with gold nanoparticles for high sensitivity mercury detection

**DOI:** 10.1038/s41598-021-89045-2

**Published:** 2021-05-04

**Authors:** Maeve H. S. McLaughlin, Alexander C. Pakpour-Tabrizi, Richard B. Jackman

**Affiliations:** London Centre for Nanotechnology and the Department of Electronic and Electrical Engineering, University College London (UCL), 17-19 Gordon Street, London, WC1H 0AH UK

**Keywords:** Materials for devices, Sensors and biosensors, Materials science, Chemistry, Electrochemistry, Materials chemistry

## Abstract

This work compares the electrochemical impedance response of polished and unpolished boron doped diamond (BDD) electrodes, during mercury detection measurements. For each substrate type both bare electrodes and electrodes decorated with average diameter 30 nm AuNPs were used, to investigate the role of AuNPs during mercury sensing with diamond electrodes. In square wave anodic stripping voltammetry (SWASV) measurements for mercury detection, the mercury ions in the electrolyte are deposited onto, then stripped from the diamond electrode surface. To investigate the different electrode performances during these steps, the EIS measurements were made at the deposition and stripping potentials, alongside scans at open circuit potential for comparison. The performance of the electrodes is assessed in terms of their electron transfer rate (k_0_). The electrodes decorated with AuNPs are shown to have lower capacitance and higher reactivity than the bare pBDD and BDD electrodes, until the mercury concentration in the electrolyte is < 500 µM, when the sp^2^/sp^3^ carbon ratio at the surface of the electrodes has a greater influence on the sensitivity for mercury detection than the presence of AuNPs.

## Introduction

Mercury is a highly toxic heavy metal that poses a severe threat to the environment and human health^1^. It is essential that mercury is detected in the environment with high sensitivity, as it tends to form complexes with biological ligands, which leads to accumulation in the food chain^2^. The primary source of mercury exposure in humans is from food, predominantly fish, which have been exposed to mercury contaminated water^3^. To detect mercury with sufficient sensitivity, electrochemical techniques are ideal, as they can be performed using portable equipment, for real-time monitoring at the site of suspected mercury contamination. The use of electrochemical impedance spectroscopy (EIS) offers some advantages over other electrochemical approaches. EIS is a steady-state technique, meaning that signal averaging can be used to achieve the required precision level within an individual experiment. With standard instrumentation, EIS measurements are conducted over a wide frequency range (< 1 MHz to > 1 MHz), which permits a broad range of electrochemical processes to be investigated in the same experiment^4^. These characteristics surpass those of equivalent techniques based on time domain experimentation and have led to EIS becoming one of the principal methods for investigating interfacial reaction mechanisms.

Whilst diamond, most typically grown by microwave plasma-enhanced chemical vapour deposition methods, is considered a wide band gap semiconductor, it can display quasi-metallic properties if boron is incorporated at concentrations in excess of 10^20^ cm^−3^^5^. Such heavily boron doped diamond (BDD) as electrode materials are well suited to the high sensitivity detection of mercury and other species via electrochemical measurements. This material is associated with low background currents, a wide electrochemical window and chemical stability^6^. In addition, BDD electrodes are stable at extreme temperatures and pressures and are resistant to fouling, so are ideal for the application of portable sensors for in situ measurements over extended periods of time, even in harsh environments^7^.

The EIS measurements in this paper are an extension to previous work by the authors, where BDD electrodes, some decorated with gold nanoparticles, were used to detect mercury in a 0.1 M HNO_3_ electrolyte via square wave anodic stripping voltammetry (SWASV)^8^. The gold nanoparticles act catalytically during the measurements, improving the sensitivity of the BDD electrodes. During the SWASV measurements the mercury in the electrolyte was pre-concentrated onto the working electrode surface by application of 0.35 V for 10 min^8,9^. Mercury was detected in the potential range 0.5–0.8 V during these SWASV measurements.

In this study, EIS measurements are used to identify the change in the reactivity of BDD-based electrodes during the different stages of the SWASV measurements described above^8^. The reactivity of the electrodes is indicated by the charge transfer resistance at their surface and quantified by an electron transfer rate (k_0_), which is calculated from the raw EIS data. The EIS measurements were performed following the application of potentials corresponding to the steps of the SWASV measurements^8^. First, an open circuit potential was applied, to serve as a control for the behaviour of the system. The pre-concentration step of the SWASV measurements was then performed, before the next set of EIS measurements. Finally, the EIS measurements were repeated after application of a potential higher than the range in which mercury is detected electrochemically (0.5–0.8 V), at which point all of the mercury ions which were pre-concentrated onto the electrode surface will have been stripped away.

## Experimental methods

Electrochemical grade BDD ([B] > 10^20^ cm^−3^, 10 × 10 × 0.5 mm) substrates were purchased from Element Six Ltd (e6cvd.com). The experiments used unpolished polycrystalline BDD, with a surface roughness R_A_ ~ 50 μm, and mechanically polished polycrystalline diamond (pBDD), where the surface roughness was reduced to R_A_ values around 50 nm (substrate thickness reduced to 0.4 mm). All chemicals, unless otherwise stated, were purchased from Sigma-Aldrich. Reverse osmosis derived water, resistivity 18 MΩ-cm, was used throughout.

### Electrode preparation

Prior to processing, organic contaminants were removed from the BDD and pBDD surfaces with a ‘Piranha’ clean (3:1 v/v of 98% HCl and 30% H_2_O_2_) for 10 min^10^.

The graphitic carbon content in the surface of each BDD and pBDD substrate was qualitatively assessed with a Renishaw inVia micro-Raman spectrometer (150 mW power, 532 nm laser source, ~ 1.5 µm spot size). The Raman analysis was performed with 20 × magnification, 10 s exposure and an average was taken over ten accumulations, the microscope was calibrated using a silicon substrate. WiRE (v 2.0) software was used for data acquisition.

It has previously been shown that gold nanoparticles (AuNPs) have stronger adherence to hydrophobic surfaces and specifically to BDD surfaces when they are hydrogen terminated, compared to when they are oxygen terminated^11,12^. For this reason the BDD and pBDD substrates were hydrogen terminated in an AX5010 Seki Technotron Inc. reactor with H-plasma for 10 min at 700 °C platen temperature (Williamson Dual wavelength pyrometer), 800 W power, 40 Torr pressure. A non-continuous 5 nm gold film was sputtered onto one each of the BDD and pBDD electrodes with an Emscope SC500 gold sputter coater. The gold films were segregated into gold nanoparticles (AuNPs) by an annealing process (often refered to as ‘de-wetting’, due to the role of surface tension) in a Solaris 150 Rapid Thermal Processing System, under nitrogen at 400 °C for 5 min.

A Zeiss XB1540 Crossbeam scanning electron microscope (10 kV operation voltage) was used to quantify the size and dispersion of AuNPs on the electrode surfaces. Scans from five random locations across each substrate were analysed with ImageJ software to calculate the average size and surface coverage of the AuNPs on each electrode.

### EIS measurements

The EIS measurements were made with a three-electrode setup, controlled by a Metrohm Autolab PGSTAT204 potentiostat, using the FRA32M EIS module, and NOVA 2.1 software. A BDD-based working electrode, with 0.14 cm^2^ surface area exposed to the electrolyte, a Ag/AgCl KCL (3 M) reference electrode and a platinum counter electrode with 1 cm^2^ surface area were used. Electrical contact from the potentiostat to the BDD electrode was made through firm contact to copper tape which was clamped under the base of the BDD electrode, with a Viton o-ring clamped onto the top surface, as has previously been shown to be effective^13^. The experiments were conducted in a 0.1 M HNO_3_ electrolyte, which was sequentially doped with increasing concentrations of mercury nitrate, from 1 pM to 1 mM Hg(NO_3_)_2_. Four BDD-based electrodes were used, a bare BDD electrode, a BDD electrode decorated with 30 ± 14 nm AuNPS, a bare pBDD electrode, and a pBDD electrode decorated with 30 ± 11 nm AuNPs.

At each concentration of mercury, the EIS measurements were performed after the application of each of three potentials for 10 min: an open circuit potential control, a deposition potential of 0.35 V, at which the mercury ions in the electrolyte are pre-concentrated onto the surface of the BDD-based working electrode and a stripping potential of 1.0 V, at which all of the pre-concentrated mercury will have been stripped from the surface of the BDD-based working electrode. After the application of each of these potentials for 10 min, an EIS measurement was performed over the frequency range 50 kHz to 50 MHz, with 8 points per decade and 10 mV amplitude. Between each addition of mercury nitrate the working electrode was cleaned by the application of 150 current pulses, lasting 100 ms, which alternated between 10 mA cm^−2^ and − 10 mA cm^−2^, as has been previously shown to be effective^14^.

EIS Spectrum Analyser software (ABC Chemistry) was used to fit the EIS results using equivalent circuit modelling. The quality of the fit for each set of experimental data is defined by a χ^2^ value, with the quality of each fit below the limit χ^2^ < 0.1. The parameters of each equivalent circuit are quantified and used to calculate the electron transfer rate (k_0_) at each electrode.

## Results

### Electrode characterisation

**Figure 1:**
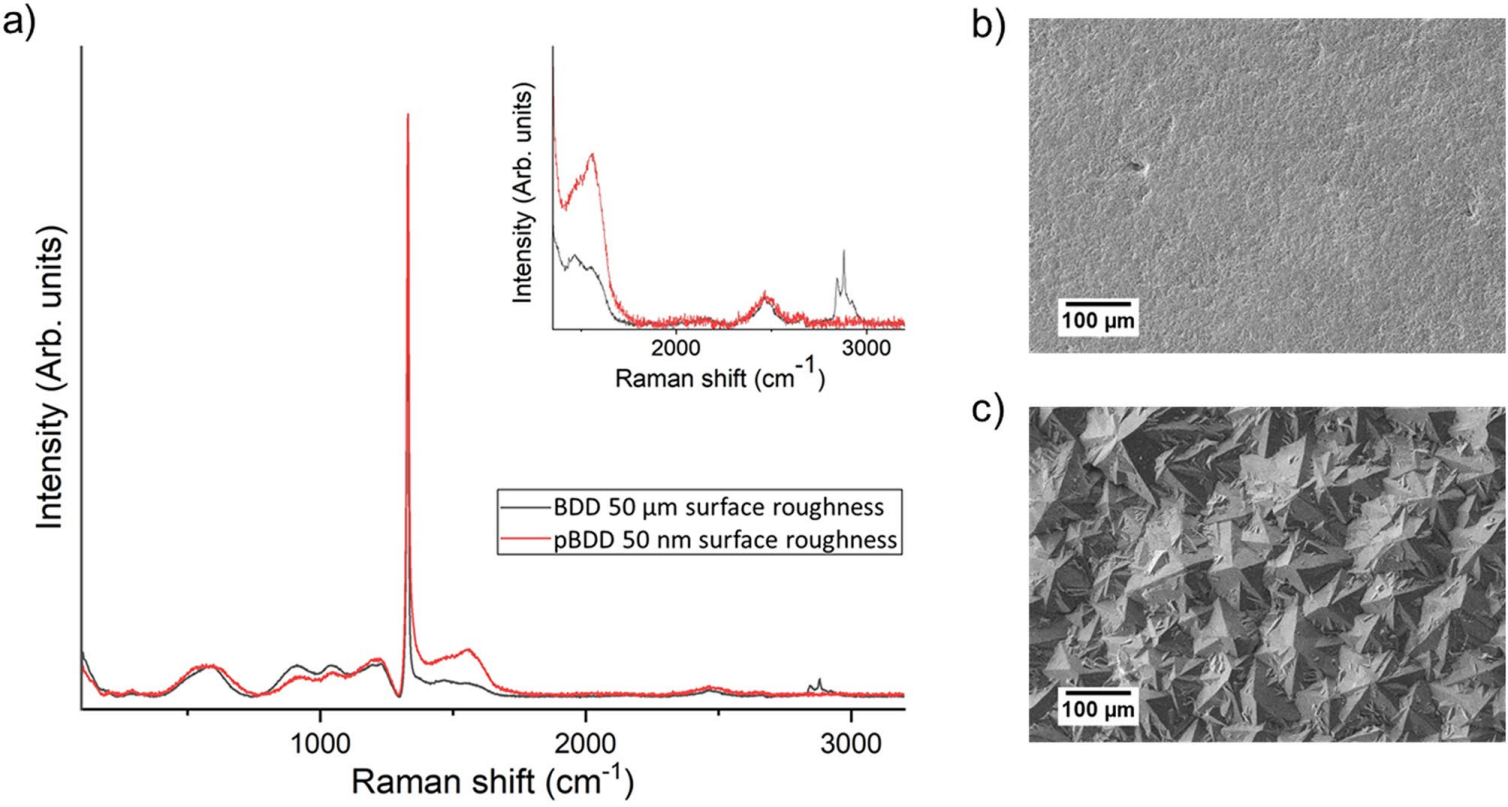
**(a)** Raman spectra of the unpolished BDD substrate revealing the distinctive 1332 cm^-1^ peak of the diamond carbon phase, and (inset) second order Raman spectrum (black) and similar measurements for the polished pBDD substrate (red), with the corresponding SEM images of **(b)** the BDD substrate and **(c)** the polished pBDD substrate.

The relative proportions of sp^3^ diamond carbon and sp^2^ non-diamond carbon at the surface of the BDD and pBDD electrodes was assessed with Raman spectroscopy (Fig. 1a). Visible Raman spectroscopy is known to overemphasise the size of the sp^2^ origin peaks as compared to those derived from the sp^3^ phase, due to the differing cross sections for Raman excitation of the two carbon phases^15^. It is clear from Fig. 1a that both films are predominantly diamond (sp^3^) in character. Crystalline quality can be estimated from the full width at half maximum (FWHM) of the 1332 cm^−1^ sp^3^ peak; in this case the values of 8.39 ± 0.11 cm^−1^ and 12.10 ± 0.18 cm^−1^ for the BDD and pBDD substrates respectively, verify the relatively high diamond crystalline quality despite the presence of very high levels of the boron dopant^16^. Figure 1b,c reveal the morphological differences between the polished and unpolished samples using SEM analysis.

**Figure 2:**
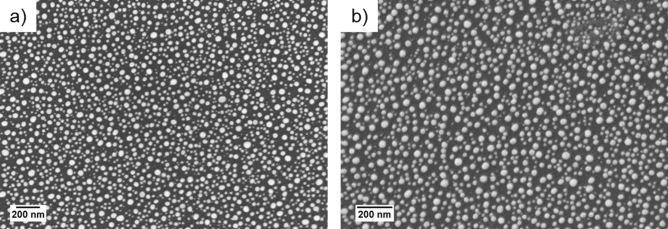
SEM images of **(a)** the BDD substrate decorated with AuNPs, average diameter 30 nm, and **(b)** the pBDD substrate decorated with AuNPs, average diameter 30 nm. The SEM images were obtained from a Zeiss XB1540 Crossbeam scanning electron microscope (10 kV operation voltage) and analysed with ImageJ sofware.

The average size and percentage coverage of the AuNPs produced on the BDD and pBDD substrates, by the annealing process described in “Electrode preparation”, were calculated using ImageJ to analyse the SEM images of each surface (Fig. 2). An average value was calculated from the size and surface coverage of the AuNPs on each electrode, from five scans at random locations on the substrate surfaces. On the BDD substrate, the average AuNP diameter was 30 ± 14 nm, with a percentage coverage of 44%. On the pBDD substrate, the average AuNP diameter was 30 ± 11 nm, with a percentage of 42%.

### EIS measurements

**Figure 3:**
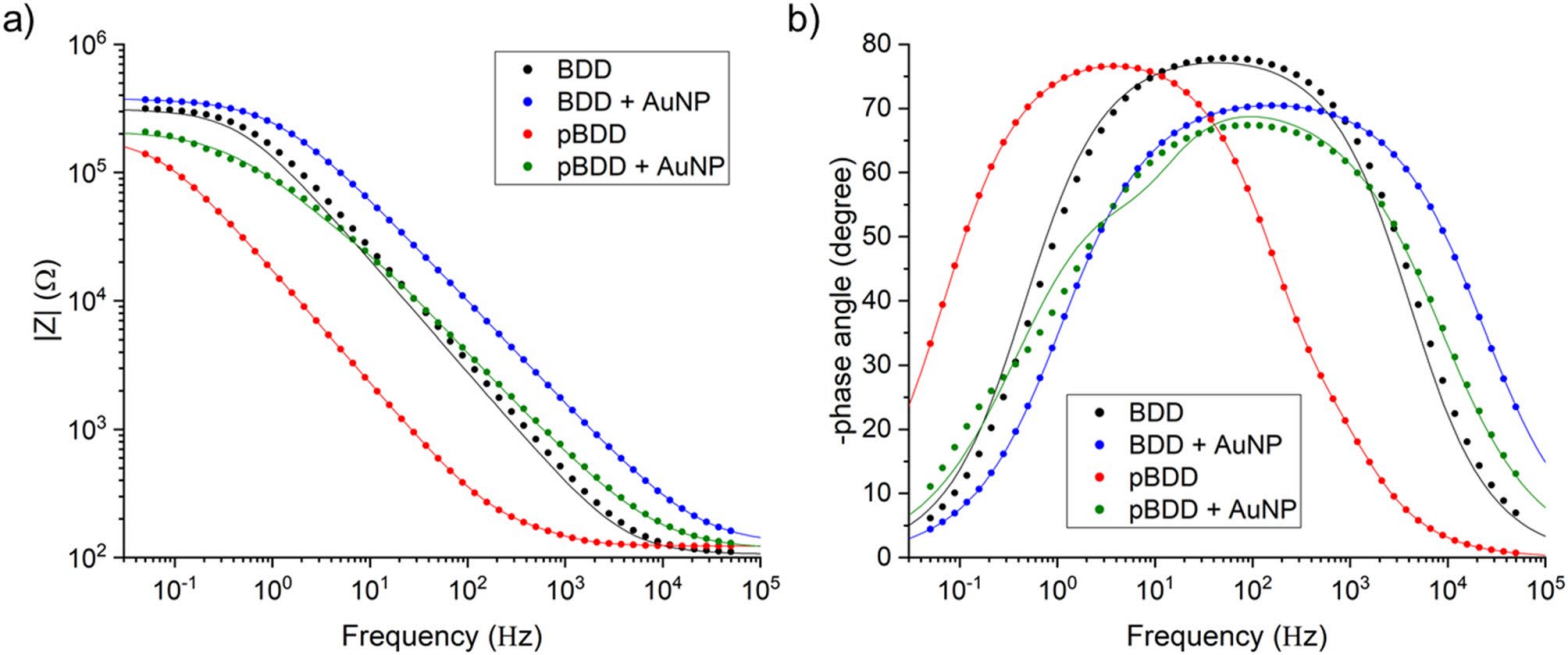
**(a)** The Bode impedance plots and **(b)** the Bode phase plots of the data for each electrode resulting from an EIS measurement performed at a mercury concentration of 1 mM, applied potential 0.35 V. The EIS measurement was conducted over the frequency range 50 kHz to 50 MHz, with 8 points per decade and 10 mV amplitude.

The Bode impedance and phase plots for each electrode are consistent under all configurations of mercury concentration and potential at which the EIS measurements were made. An example of these plots, made at the deposition potential, 0.35 V, when the mercury concentration in the electrolyte was 1 mM, is shown in Fig. 3. The Bode plots for each electrode in the control blank electrolyte and over the full range of mercury concentrations and potentials tested are provided in the Supplementary Information.

**Figure 4:**
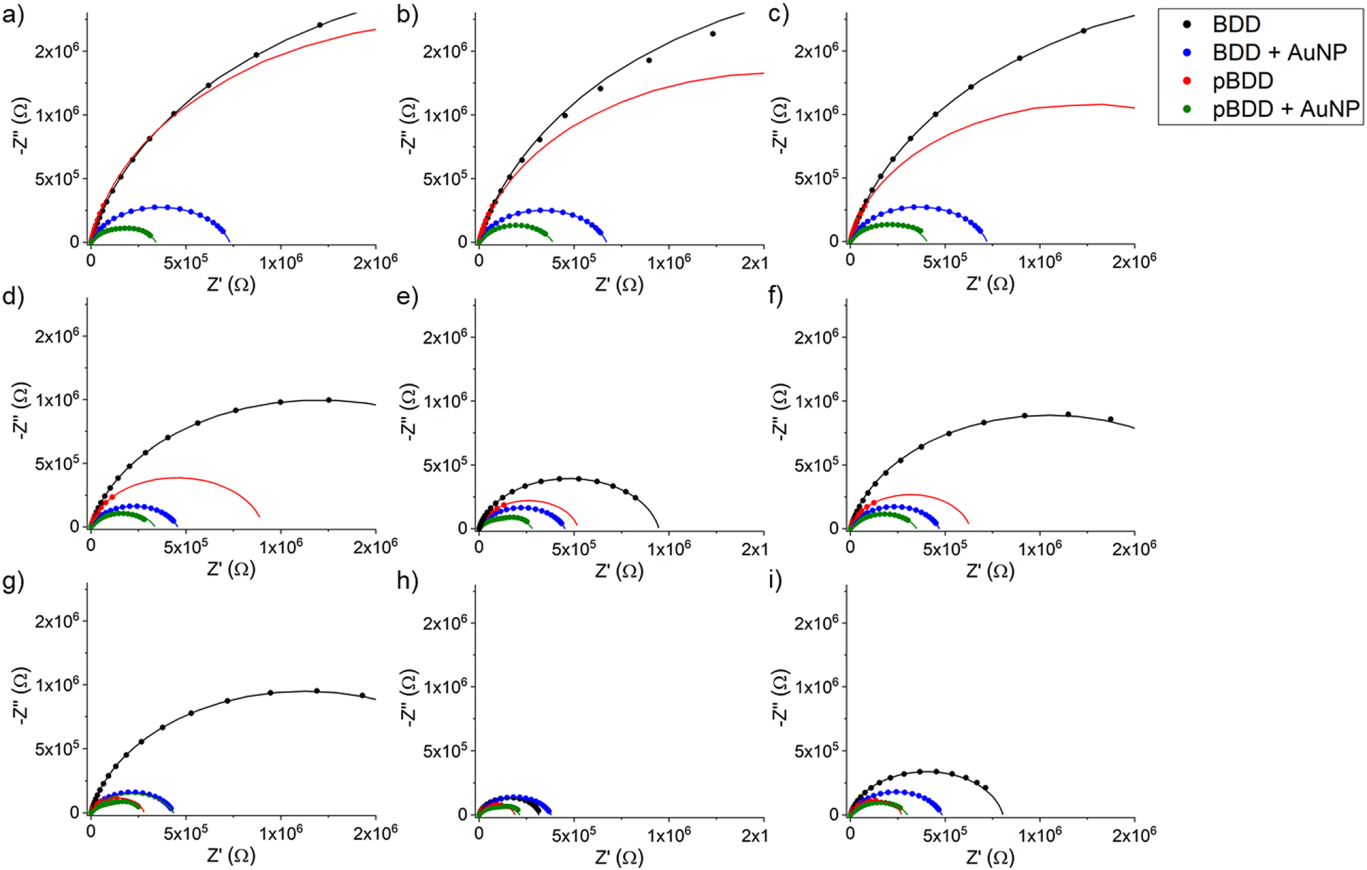
Nyquist plot presentation of the EIS data from measurements made over the frequency range 50 kHz to 50 MHz, with 8 points per decade and 10 mV amplitude, for each of the electrodes. The results presented here are from EIS measurements made at **(a)** open circuit potential, 1 µM Hg concentration, **(b)** deposition potential (0.35 V), 1 µM Hg concentration, **(c)** stripping potential (1.0 V), 1 µM Hg concentration, **(d)** open circuit potential, 500 µM Hg concentration, **(e)** deposition potential (0.35 V), 500 µM Hg concentration, **(f)** stripping potential (1.0 V), 500 µM Hg concentration, **(g)** open circuit potential, 1 mM Hg concentration, **(h)** deposition potential (0.35 V), 1 mM Hg concentration and **(i)** stripping potential (1.0 V), 1 mM Hg concentration. The Nyquist plots for the other mercury concentration and potentials tested with EIS are presented in the Supplementary Information.

The EIS results for each of the electrodes, at the mercury concentrations: 1 µM, 500 µM, and 1 mM, are presented in the Nyquist plot format in Fig. 4. In the frequency range used here (50 kHz to 50 MHz), which is standard for the analysis of Faradaic processes in aqueous systems with EIS, only one semicircle is seen on the Nyquist plots^17^. The Nyquist plots for the EIS measurements made in the control blank electrolyte and the other mercury concentrations tested (1 pM and 1 nM) are presented in the Supplementary Information.

**Figure 5:**
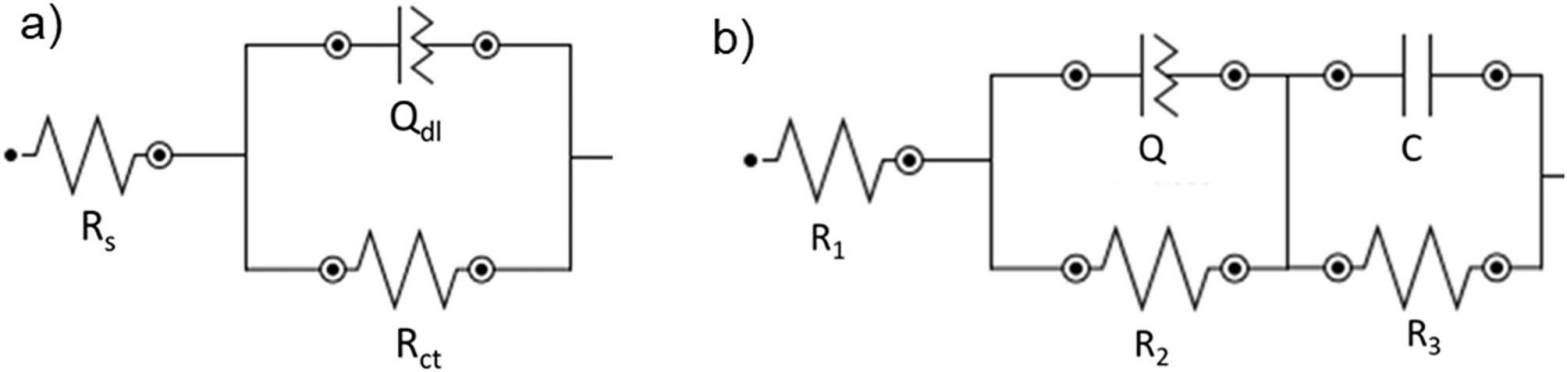
**(a)** The equivalent circuit used to model the EIS data for the BDD electrode and the BDD electrode decorated with 30 ± 14 nm AuNPs and **(b)** the equivalent circuit model for the pBDD electrode and the pBDD electrode decorated with 30 ± 11 nm AuNPs.

The equivalent circuit models that were used to make the fits to the Bode and Nyquist plots in Figs. 3 and 4 are presented in Fig. 5. The fits of the equivalent circuits to the raw data all fit the criteria of χ^2^ < 0.1. In both of the equivalent circuit models (Fig. 5) a constant phase element (Q) has been used to represent the double layer capacitance at the surface of the diamond electrodes, as is convention for rough electrode surfaces and polycrystalline materials^18^. The impedance of Q is calculated using Eq. (1), in which Y_0_ is a constant, *j* is the imaginary number √−1, ω is the angular frequency and N is the exponent (0–1).

1 $${Z}_{Q}= \frac{1}{{Y}_{0}(j{)}^{N}}$$

Q is used to calculate the effective capacitance using Eq. (2), in which C_eff_ is the effective capacitance, R_s_ is the solution resistance and R_ct_ is the charge transfer resistance at the diamond-based electrode.

2 $${C}_{eff}={{Y}_{0}}^\frac{1}{2}\cdot [\frac{1}{{R}_{s}}+ \frac{1}{{R}_{ct}}{]}^{\frac{N-1}{N}}$$

The BDD and BDD + AuNP electrodes are modelled with a modified simple Randles equivalent circuit, made up of a series resistor (R_s_) to represent the solution resistance of the electrolyte, a parallel resistor (R_ct_) to represent the charge transfer resistance at the surface of the diamond electrodes, and a constant phase element (Q_dl_), which represents the capacitance of the double layer at the surface of the diamond electrodes (Fig. 5a). An additional RC circuit is added in parallel for the equivalent circuit designed to model the EIS data for the pBDD and pBDD + AuNP electrodes. This is necessary due to the higher proportion of sp^2^ carbon at the surface of these electrodes, which has been shown to have a significant effect on the electrode reaction kinetics of some redox systems^19,20^. The additional parallel resistor (R_3_) represents the charge transfer resistance at the sp^2^ carbon regions on the surface of the pBDD-based electrodes and the parallel capacitor (C) represents the double layer capacitance at the sp^2^ carbon regions of the pBDD-based electrode surfaces (Fig. 5b).

The parameters extracted from the equivalent circuit fits for the EIS results at open circuit potential of the BDD-based and pBDD-based electrodes are presented in Tables 1 and 2 respectively. The parameters extracted from the equivalent circuit fits of the EIS results conducted at the deposition potential (0.35 V) and the stripping potential (1.0 V) are provided in the Supplementary Information.

**Table 1:** The parameters extracted from the equivalent circuit (Fig. 5a) fit of the raw EIS data for the BDD and BDD + AuNP electrodes at open circuit potential.

Electrode	Hg concentration	R_s_ (Ω)	R_ct_ (MΩ)	C_eff_ (µF/cm^2^)	χ^2^
BDD	0 M (control)	38.1	1.61	2.14	0.07
BDD + AuNP	0 M (control)	36.8	0.52	0.36	0.10
BDD	1 pM	61.1	2.89	2.14	0.02
BDD + AuNP	1 pM	86.8	0.69	0.36	0.03
BDD	1 nM	87.7	4.69	2.21	0.01
BDD + AuNP	1 nM	105.0	0.70	0.43	0.02
BDD	1 µM	99.6	4.97	2.21	0.01
BDD + AuNP	1 µM	123.0	0.73	0.43	0.01
BDD	500 µM	107.0	2.41	2.29	0.03
BDD + AuNP	500 µM	121.0	0.46	0.36	0.04
BDD	1 mM	126.0	0.98	2.07	0.06
BDD + AuNP	1 mM	129.0	0.44	0.43	0.03

The parameters extracted from the equivalent circuit fit of the EIS data for these electrodes at the other potentials tested are presented in the Supplementary Information.

**Table 2:** The parameters extracted from the equivalent circuit (Fig. 5b) fit of the raw EIS data for the pBDD and pBDD + AuNP electrodes at open circuit potential.

Electrode	Hg concentration	R_1_ (Ω)	R_2_ (MΩ)	R_3_ (kΩ)	C_eff_ (µF/cm^2^)	C (µF/cm^2^)	χ^2^
pBDD	0 M (control)	46.7	0.38	0.02	25.57	67.14	0.03
pBDD + AuNP	0 M (control)	21.8	0.51	1.76	0.50	18.86	0.04
pBDD	1 pM	71.3	2.24	0.03	30.29	49.5	0.08
pBDD + AuNP	1 pM	60.3	0.38	1.98	0.71	14.79	0.03
pBDD	1 nM	87.8	3.11	0.04	31.21	46.71	0.08
pBDD + AuNP	1 nM	87.8	0.42	2.16	0.71	14.43	0.02
pBDD	1 µM	107.0	4.01	0.04	33.00	45.36	0.07
pBDD + AuNP	1 µM	111.0	0.33	1.89	0.71	14.71	0.04
pBDD	500 µM	116.0	0.91	0.04	33.14	50.43	0.03
pBDD + AuNP	500 µM	109.0	0.32	3.85	0.21	17.36	0.06
pBDD	1 mM	123.0	2.80	0.03	34.14	52.64	0.06
pBDD + AuNP	1 mM	115.0	0.27	5.70	0.93	14.14	0.07

The parameters extracted from the equivalent circuit fit of the EIS data for these electrodes at the other potentials tested are presented in the Supplementary Information.

The electron transfer rate, k_0_, was calculated using Eq. (3), in which R is the universal gas constant, T is the absolute temperature, F is Faraday’s constant, n is the number of electrons transferred, S is the surface area of the working electrode exposed to the electrolyte and C_0_ is the concentration of mercury in the electrolyte.

3 $${k}_{0}=\left(\frac{RT}{nF}\right)\cdot \frac{1}{nSF{R}_{ct}{C}_{0}}$$

The k_0_ values calculated from the parameters extracted from the equivalent circuit modelling of each of the electrodes, from the EIS measurements over the Hg concentration range 1 pM to 1 mM are displayed in Table 3.

**Table 3:** The electron transfer rates of each of the electrodes tested at 1 pM mercury concentration, calculated from the parameters extracted from equivalent circuit modelling fits of the raw EIS data (see Supplementary Information).

Electrode	k_0_ at open circuit potential (cm s^−1^)	k_0_ at deposition potential (0.35 V) (cm s^−1^)	k_0_ at stripping potential (1.0 V) (cm s^−1^)
BDD	0.16 ± 0.03	0.16 ± 0.03	0.14 ± 0.03
BDD + AuNP	0.67 ± 0.03	1.20 ± 0.03	0.94 ± 0.03
pBDD	0.21 ± 0.03	0.21 ± 0.03	0.22 ± 0.03
pBDD + AuNP	1.24 ± 0.03	1.08 ± 0.03	1.09 ± 0.03

## Discussion

The relative proportions of sp^3^ diamond carbon and sp^2^ non-diamond carbon at the surfaces of the BDD and pBDD substrates were assessed with Raman spectroscopy (Fig. 1). The characteristic 1332 cm^−1^ diamond carbon peak is clearly displayed in the Raman spectra for both substrate types. The non-diamond carbon G peak at 1575 cm^−1^ is also present in both spectra, but at greater intensity in the spectra for the pBDD substrate. The ratio between the intensities of the 1332 cm^−1^ and 1575 cm^−1^ peaks can only be used to qualitatively assess the relative proportions of these two carbon bond types. It is not possibly to quantify this ratio because the intensity of each peak is dependent on the grain size, film stress, doping density, and excitation wavelength used^21^. When the excitation wavelength is in the visible spectrum, as in this paper where a 532 nm laser source was used, the sensitivity to sp^2^ materials is approximately 100 × higher than for sp^3^ material^22^.

As mentioned previously, the excitation wavelength used here has the effect of emphasisng the sp^2^ carbon bond strength in comparison to the sp^3^ diamond peak. Despite this, the 1575 cm^−1^ peak representative of sp^2^ carbon is very low intensity compared to the 1332 cm^−1^ sp^3^ carbon peak in these Raman spectra, indicating that the near surface region of these samples is relatively low in sp^2^ content. The intensity of the 1575 cm^−1^ peak is higher relative to the 1332 cm^−1^ peak for the pBDD substrate, meaning that there is a higher proportion of non-diamond carbon at the surface of this substrate. This is likely due to the polishing process introducing damage to the diamond surface. It has previously been shown that mechanical polishing causes some micro-cracks to form at the surface of polished diamond, which can extend several µm into the substrate and result in surface stable sp^2^ carbon^23^.

The high boron doping concentration in the BDD and pBDD substrates ([B] > 10^20^ cm^−3^) is confirmed by the asymmetry at the base of the 1332 cm^−1^ peak, known as the Fano resonance, which corresponds to the onset of metal-like conductivity in the diamond as a result of the boron impurity band transitioning into a continuum state^24^. The broad peaks between 500 cm^−1^ and 1200 cm^−1^ in both Raman spectra (Fig. 1) further indicate the high boron doping concentration in the substrates, greater than 10^20^ boron atoms cm^−3^ within the diamond structures^25^.

The raw EIS data is presented in the Bode plot format in Fig. 3. In the high frequency region of the Bode impedance plots (Fig. 3a) a horizontal amplitude is reached and in the high frequency region of the Bode phase plots (Fig. 3b) the phase angle tends to 0°. These responses are typical of uncompensated resistance, which in this experiment will predominantly be due to the solution resistance through the electrolyte (R_s_). In the low frequency region of the Bode impedance plots the curve is horizontal and the impedance is therefore independent of frequency, which corresponds to the total impedance of the system. In the middle frequency region, the Bode impedance plots are linear (slope close to −1) and the phase angle tends to −90° in the Bode phase plots. These characteristics are typical of a capacitor, which in this system is due to the double layer capacitance at the surface of the diamond-based working electrodes^26^.

The Nyquist plots in Fig. 4 show that generally, the bare diamond electrodes (both BDD and pBDD) have significantly higher charge transfer resistance (R_ct_) than the electrodes decorated with AuNPs. This means that the bare electrodes will be slower to respond to a change in mercury concentration in the solution than the electrodes coated with AuNPs. The semicircle on the Nyquist plots for the bare BDD electrode are the highest and correspond to the highest R_ct_ at the surface of that electrode. This is because the higher proportion of sp^2^ carbon at the surface of the pBDD-based electrodes and the presence of the gold nanoparticles on the decorated electrodes improves their reactivity, so these electrodes will have lower R_ct_ than the bare BDD electrode^8,11^.

The Nyquist plots for the EIS data recorded for the mercury concentrations between 1 pM and 1 µM are consistent and very similar to the Nyquist plots for the data recorded during the control measurements in the blank electrolyte, when no mercury was present (see Supplementary Information). This means that within this concentration range, each of the working electrodes tested were passive, with no change in the electron transfer rate at their surface with the increasing mercury concentration and so, no mercury would have been detected in an electrochemical measurement. Higher sensitivity was achieved by the authors in a previous paper, using SWASV to detect mercury with AuNP decorated BDD electrodes^8^. It is well established that the strength of SWASV measurements is that they are run at very low noise levels in contrast to the measurements presented here, which are unlikely to be able to reveal such sensitive measurements due to the inherently noisier nature of the processes being recorded.

When the concentration of mercury in the electrolyte was increased to 500 µM there was a significant reduction in the size of the part of the semicircle presented in the Nyquist plots, for the EIS measurements at the deposition potential (0.35 V, Fig. 4e). At this potential, the mercury ions in the solution were pre-concentrated onto the surface of the electrodes. The reduction in the size of the semicircle is due to a decrease in R_ct_ at the surface of the electrodes. R_ct_ values are smaller for the decorated electrodes, as the AuNPs act catalytically during the application of the deposition potential, causing more mercury ions to become more strongly attached the electrode surface, meaning that mercury is detected at lower concentrations when the AuNPs are present^8^. The Nyquist plot recorded at the stripping potential (1.0 V) at this concentration is similar to the one recorded at open circuit potential. This shows that all of the mercury has been stripped from the surface of the electrodes and that no further reaction is taking place at their surface.

When the concentration of mercury in the electrolyte was increased to 1 mM there was a further reduction in the size of the semicircles on the Nyquist plot recorded at the deposition potential. The R_ct_ values extracted from this plot are smaller for the pBDD-based electrodes than the BDD electrodes, whether or not the electrodes were decorated with AuNPs. Therefore, at this higher concentration of mercury the higher proportion of sp^2^ carbon at the surface of the pBDD-based electrodes also has an impact on the electron transfer kinetics, in addition to the AuNPs. It has previously been shown that the presence of sp^2^ carbon can lead to the adsorption of analytes onto the electrode surface, commonly referred to as fouling^27^. In the case of the pBDD electrodes here, it is possible that at the higher mercury concentrations, mercury is pre-concentrated onto the sp^2^ regions of the pBDD surface during the application of the deposition potential (0.35 V), which is conventionally only the case when AuNPs are present. This requires further investigation. Again, the Nyquist plot recorded at the stripping potential is similar to that recorded at an open circuit potential. However, it appears that not all of the mercury has been removed from the surface of the bare BDD electrode, as the R_ct_ value is lower than that recorded at the open circuit potential, indicating that some reaction may still be happening at this electrode surface.

The equivalent circuits used to model the electrochemical systems in this work are based on the simple Randles equivalent circuit^28^. A constant phase element was used in place of a traditional capacitor to represent the double layer capacitance at the surface of the diamond-based electrodes. This is common practise for polycrystalline materials, for which the double layer capacitance is non-homogenous across their surface^18^. An additional RC circuit was added in parallel to the modified simple Randles equivalent circuit for the pBDD-based electrodes, to more accurately model their surfaces, which have a larger proportion of sp^2^ carbon, introduced during the polishing process. The additional RC circuit models the charge transfer resistance and double layer capacitance at the sp^2^ carbon regions of these electrodes. For each electrode type, BDD or pBDD, the same equivalent circuit model could be used for the bare electrode or when decorated with AuNPs. This demonstrates that although the presence of the AuNPs improves the sensitivity of the electrodes by aiding the pre-concentration of mercury ions in the bulk electrolyte onto the electrode surfaces, the response of the mercury detection measurements is dominated by the diamond electrodes.

The resistance values extracted from the equivalent circuits fits of the EIS data for each electrode are of similar magnitudes. The significant difference between the bare and AuNP decorated electrodes of each type, BDD or pBDD, is the capacitance values. In each case, the capacitance or effective capacitance, is higher for the bare BDD and pBDD electrodes. A high quality BDD electrode, with a low concentration of sp^2^ impurities, will have a capacitance < 10 uF/cm^2^. This is the case for the BDD-based electrodes over the full mercury concentration range tested, which shows that the base substrate is a high quality BDD electrode material. Both the effective capacitance (C_eff_) at the sp^3^ regions and the capacitance (C) at the sp^2^ regions of the bare pBDD electrode are > 10 uF/cm^2^_,_ due to the larger proportion of sp^2^ carbon in its structure. The capacitance values at the sp^2^ regions of the AuNP decorated pBDD electrode are also > 10 uF/cm^2^ due to the larger proportion of sp^2^ carbon. Although, the addition of AuNPs to the surface of the pBDD electrode does reduce the effective capacitance at the sp^3^ carbon regions of its surface to < 10 uF/cm^2^. As the capacitance values are much smaller for the AuNP decorated electrodes this means that lower detection limits are possible using these electrodes, compared to the bare BDD and pBDD electrodes^7^.

The electron transfer rates (k_0_) calculated for each of the electrodes are provided in Table 3. For the bare BDD and pBDD electrodes there is little variation (± 0.02 cm s^−1^) in k_0_ at each of the potentials at which the EIS measurements were made. Therefore, the kinetics and charge transfer resistance at the surface of these electrodes did not change when the mercury was pre-concentrated onto or stripped from their surface, demonstrating that the BDD and pBDD electrodes must be decorated with gold for the pre-concentration of mercury ions from the bulk electrolyte to occur. When the electrodes are not decorated with AuNPs, the mercury ions are not pre-concentrated onto their surface, meaning that mercury will not be detected until a larger concentration has been added to the electrolyte. At each potential, the k_0_ of the AuNP decorated electrodes is higher, showing that even when the mercury ions in the electrolyte are not pre-concentrated onto the electrode surface, the decorated electrodes induce lower charge transfer resistance and faster kinetics at their surface.

## Conclusions

This work presents a detailed EIS study, investigating the role of AuNPs deposited onto the surface of diamond electrodes for electrochemical mercury detection. The systems analysed used BDD and polished pBDD electrodes, both bare and decorated with average diameter 30 nm AuNPs, for EIS measurements in a 0.1 M HNO_3_ electrolyte which was sequentially doped with increasing concentrations of mercury nitrate from 1 pM to 1 mM.

It is demonstrated that the mercury detection process is dominated by the reactivity of the diamond electrodes, as the same equivalent circuit type can be used for the bare and AuNP decorated electrodes of each substrate type (BDD and pBDD). The AuNPs improve the efficiency of the mercury detection process at each electrode, with a corresponding reduction in the R_ct_ value when they are present. The AuNPs do not change the reaction pathway at the diamond electrodes, but improve the sensitivity of the electrodes by aiding the pre-concentration of mercury ions from the bulk electrolyte, causing mercury to be detected at lower concentrations than when a bare diamond electrode is used. The electrodes decorated with AuNPs have lower capacitance and electron transfer rates, meaning that these electrodes have higher sensitivity for mercury detection than the bare BDD and pBDD electrodes.

When the electrolyte is doped with higher concentrations of mercury (> 500 µM) the relative proportions of sp^2^/sp^3^ carbon have a greater influence on the sensitivity of the electrode for mercury detection than the presence of the AuNPs. The pBDD-based electrodes, which have a higher proportion of sp^2^ carbon at their surface, are shown to have lower charge transfer resistance than the BDD-based electrodes, at higher mercury concentrations. It is suggested that at the higher concentrations of mercury the application of the deposition potential (0.35 V) possibly causes mercury ions in the bulk electrolyte to become pre-concentrated onto the sp^2^ regions of the pBDD surfaces.

The exceptional capacitance values and low k_0_ values reported, highlight how robust diamond electrodes can be optimised for high sensitivity detection. These electrodes are ideal for the development of commercial mercury sensors in aquatic environments.

## Supplementary Information

Supplementary Information.

## References

[1] Lang, D. *Guidelines for Drinking Water Qualtiy* (World Health Organization, 2017)

[2] Aragay G, Pons J, Merkoçi A (2011). Recent trends in macro-, micro-, and nanomaterial-based tools and strategies for heavy-metal detection. Chem. Rev..

[3] Järup L (2003). Hazards of heavy metal contamination. Br. Med. Bull..

[4] MacDonald, D. D. *Transient Techniques in Electrochemistry* (Plenum Press, 1977)

[5] Pierson, H. O. *Handbook of Carbon, Graphite, Diamonds and Fullerences. Properties, Precessing and Applications*. (Noyes Publications, 1994)

[6] Manivannan A, Seehra MS, Fijishima A (2004). Detection of mercury at the ppb level in solution using boron-doped diamond electrode. Fuel Process. Technol..

[7] Macpherson JV (2015). A practical guide to using boron doped diamond in electrochemical research. Phys. Chem. Chem. Phys..

[8] McLaughlin MHS (2019). Diamond electrodes for high sensitivity mercury detection in the aquatic environment: Influence of surface preparation and gold nanoparticle activity. Electroanalysis.

[9] Laffont L (2015). Mercury(II) trace detection by a gold nanoparticle-modified glassy carbon electrode using square-wave anodic stripping voltammetry including a chloride desorption step. Talanta.

[10] Nguyen CV (2016). Effect of glass surface treatments on the deposition of highly transparent reduced graphene oxide films by dropcasting method. Colloids Surf. A.

[11] Svanberg-Larsson (2016). A comparison of explicitly-terminated diamond electrodes decorated with gold nanoparticles. Electroanalysis.

[12] Ahmed SR (2017). Situ self-assembly of gold nanoparticles on hydrophilic and hydrophobic substrates for influenza virus-sensing platform. Sci. Rep..

[13] Holt KB (2004). Scanning electrochemical microscopy and conductive probe atomic force microscopy studies of hydrogen-terminated boron doped diamond electrodes with different dopin levels. J. Phys. Chem. B.

[14] Kiran R (2013). Boron doped diamond electrodes for direct measurement in biological fluids: An in situ regeneration approach. J. Electrochem. Soc..

[15] Leeds SM (1998). Use of different excitation wavelengths for the analysis of CVD diamond by laser Raman spectroscopy. Diam. Relat. Mater..

[16] Stenman F (1969). Width of the 1332-cm^−^^1^ Raman line in diamond. J. Appl. Phys..

[17] Gabrielli, C. Indentification of electrochemical processes by frequency response analysis. *Solatron Anal.* (1998).

[18] Bardini, L. *EIS 101, An Introduction to Electrochemical Spectroscopy* (2016).

[19] De Araújo DA (2014). Electrochemical conversion/combustino of a model organic pollutant on BDD anode: Role of sp^3^/sp^2^ ratio. Electrochem. Commun..

[20] Bennett JA (2004). Effect of sp^2^-bonded nanodiamond carbon impurity on the response of boron-doped polycrystalline diamond thin-film electrodes. J. Electrochem. Soc..

[21] Bormett RW, Asher SA (1995). Ultraviolet Raman spectroscopy characterizes chemical vapour deposition diamond film growth and oxidation. J. Appl. Phys..

[22] Bachmann, P. K. *et al.* Diamond and diamond-like materiall synthesis. *Mater. Res. Soc.* 99–112 (1998).

[23] Schuelke T, Grotjohn TA (2013). Diamond polishing. Diamond Relat. Mater..

[24] Ager JW, Walukiewicz W, McCluskey M (1995). Fano interference of the Raman phonon in heavily boron-doped diamond films grown by chemical vapor deposition. Appl. Phys. Lett..

[25] Prawer S, Nemanich RJ (2004). Raman spectroscopy of diamond and doped diamond. Philos. Trans. R. Soc. A Math. Phys. Eng. Sci..

[26] Petovar A, Xhanari K, Finšgar M (2018). A detailed electrochemical impedance spectroscopy study of a bismuth-film glassy carbon electrode for trace metal analysis. Anal. Chim. Acta.

[27] Garcia-Segura S, dos Santos EV, Martínez-Huitle CA (2015). Role of sp^3^/sp^2^ ratio on the electrocatalytic properties of boron doped diamond electrodes: A mini review. Electrochem. Commun..

[28] Randviir, E. The application of electrochemical impedance spectroscopy to electrochemical sensor devices. *SPR Electrochem.* (2019).

