# Supplementary material for: A detailed EIS study of boron doped diamond electrodes decorated with gold nanoparticles for high sensitivity mercury detection
Source: Sci Rep. 2021 May 4;11:9505. doi: 10.1038/s41598-021-89045-2 (PMC8096807; doi:10.1038/s41598-021-89045-2)
Supplement: Supplementary file 1 — Supplementary Information. [file 41598_2021_89045_MOESM1_ESM.docx]

Supplementary Information

A detailed EIS study of boron doped diamond electrodes decorated with gold nanoparticles for high sensitivity mercury detection

Maeve H. S. McLaughlin, Alexander C. Pakpour-Tabrizi, Richard B. Jackman^*^

London Centre for Nanotechnology and Department of Electronic and Electrical Engineering, University College London (UCL), 17-19 Gordon Street, London, WC1H 0AH, UK.

^*^Author for correspondence, e-mail: [r.jackman@ucl.ac.uk](mailto:r.jackman@ucl.ac.uk)


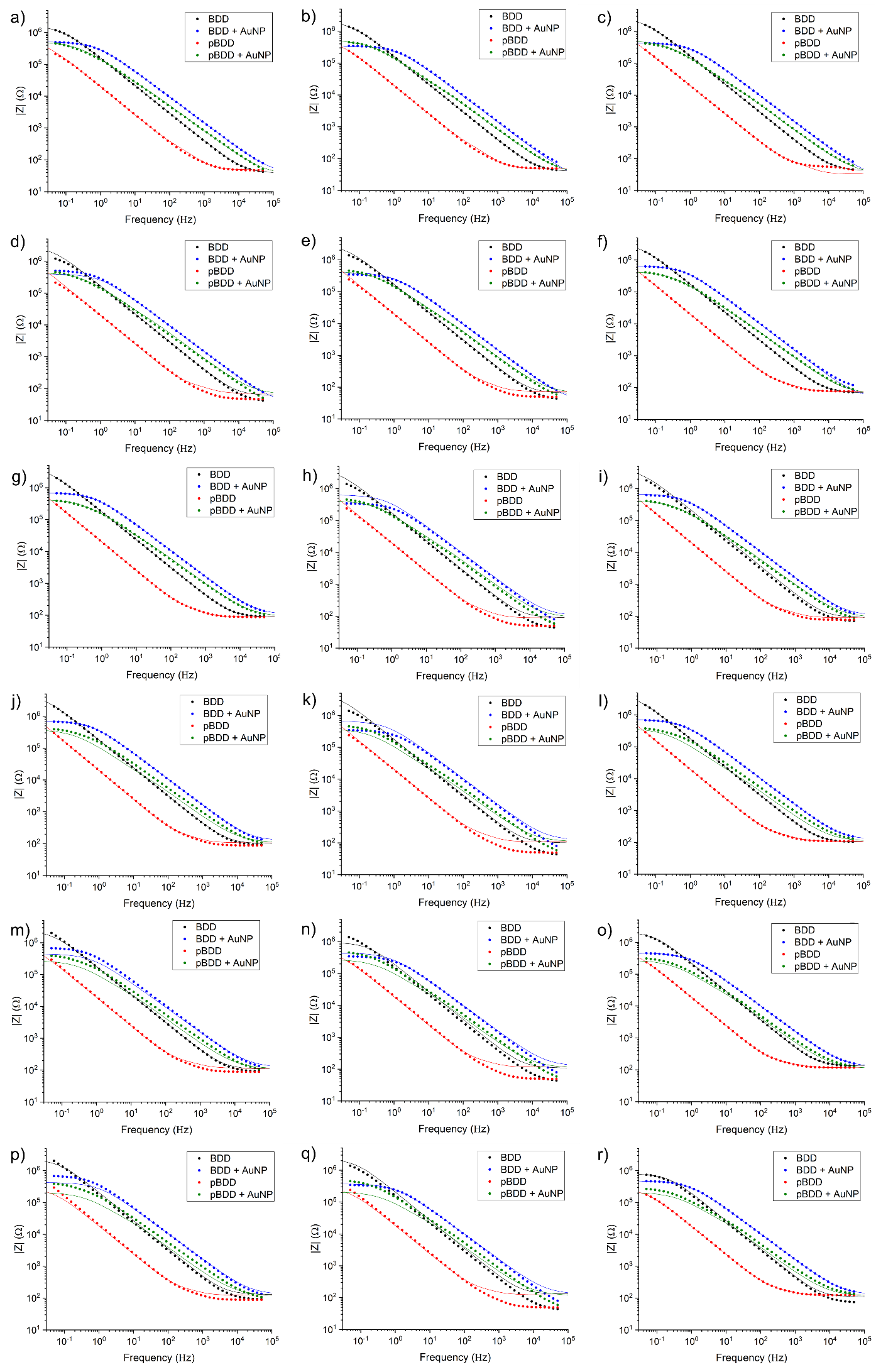


Bode impedance plot presentation of the EIS data from measurements made over the frequency range 50 kHz – 50 mHz, with 8 points per decade and 10 mV amplitude, for each of the electrodes. The results presented here are from EIS measurements made at a) open circuit potential, 0 M Hg concentration, b) deposition potential (0.35 V), 0 M Hg concentration, c) stripping potential (1.0 V), 0 M Hg concentration, d) open circuit potential, 1 pM Hg concentration, e) deposition potential (0.35 V), 1 pM Hg concentration, f) stripping potential (1.0 V), 1 pM Hg concentration, g) open circuit potential, 1 nM Hg concentration, h) deposition potential (0.35 V), 1 nM Hg concentration and i) stripping potential (1.0 V), 1 nM Hg concentration, at j) open circuit potential, 1 µM Hg concentration, k) deposition potential (0.35 V), 1 µM Hg concentration, l) stripping potential (1.0 V), 1 µM Hg concentration, m) open circuit potential, 500 µM Hg concentration, n) deposition potential (0.35 V), 500 µM Hg concentration, o) stripping potential (1.0 V), 500 µM Hg concentration, p) open circuit potential, 1 mM Hg concentration, q) deposition potential (0.35 V), 1 mM Hg concentration and r) stripping potential (1.0 V), 1 mM Hg concentration.


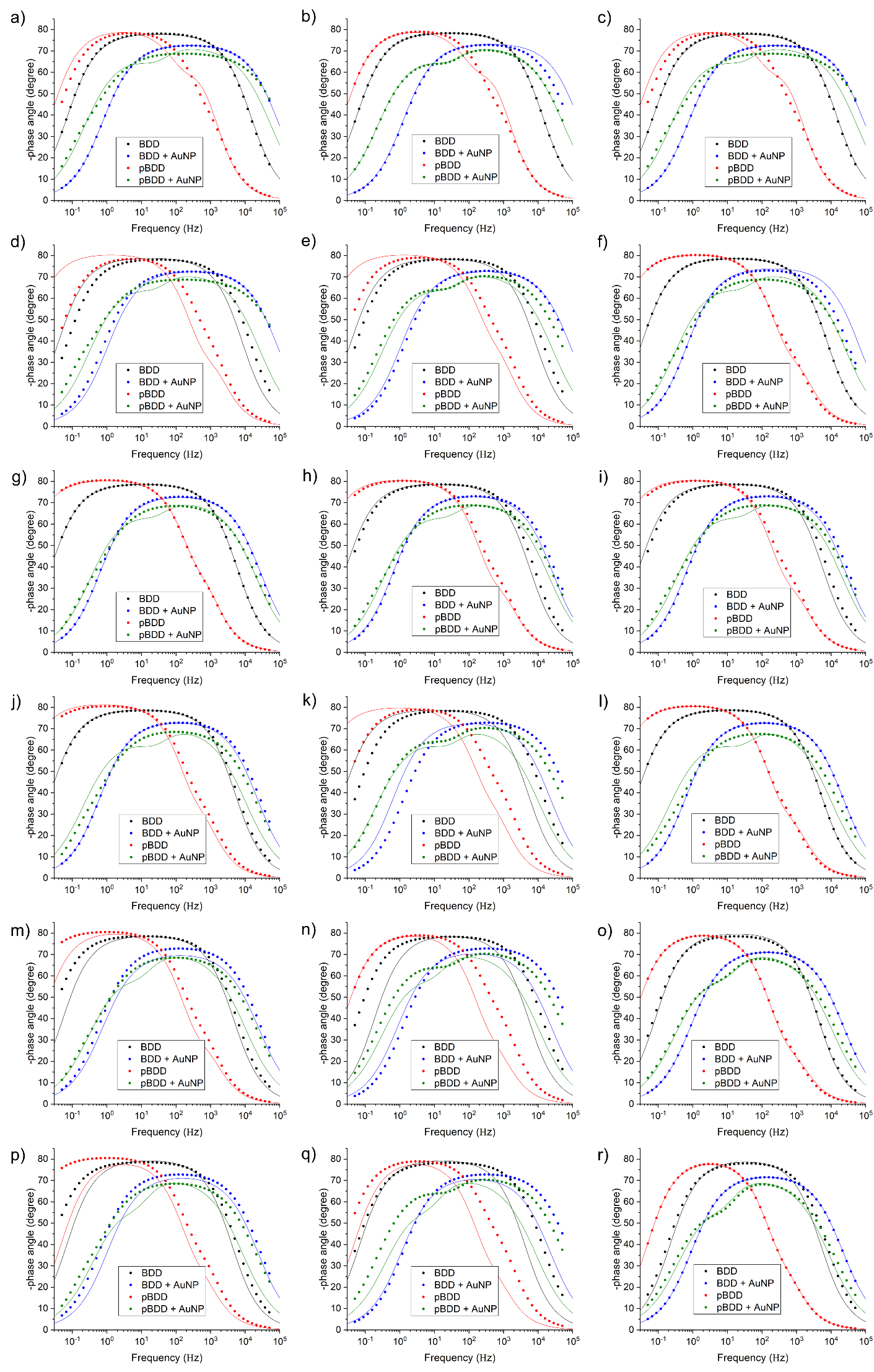


Bode phase plot presentation of the EIS data from measurements made over the frequency range 50 kHz – 50 mHz, with 8 points per decade and 10 mV amplitude, for each of the electrodes. The results presented here are from EIS measurements made at a) open circuit potential, 0 M Hg concentration, b) deposition potential (0.35 V), 0 M Hg concentration, c) stripping potential (1.0 V), 0 M Hg concentration, d) open circuit potential, 1 pM Hg concentration, e) deposition potential (0.35 V), 1 pM Hg concentration, f) stripping potential (1.0 V), 1 pM Hg concentration, g) open circuit potential, 1 nM Hg concentration, h) deposition potential (0.35 V), 1 nM Hg concentration and i) stripping potential (1.0 V), 1 nM Hg concentration, at j) open circuit potential, 1 µM Hg concentration, k) deposition potential (0.35 V), 1 µM Hg concentration, l) stripping potential (1.0 V), 1 µM Hg concentration, m) open circuit potential, 500 µM Hg concentration, n) deposition potential (0.35 V), 500 µM Hg concentration, o) stripping potential (1.0 V), 500 µM Hg concentration, p) open circuit potential, 1 mM Hg concentration, q) deposition potential (0.35 V), 1 mM Hg concentration and r) stripping potential (1.0 V), 1 mM Hg concentration.

**
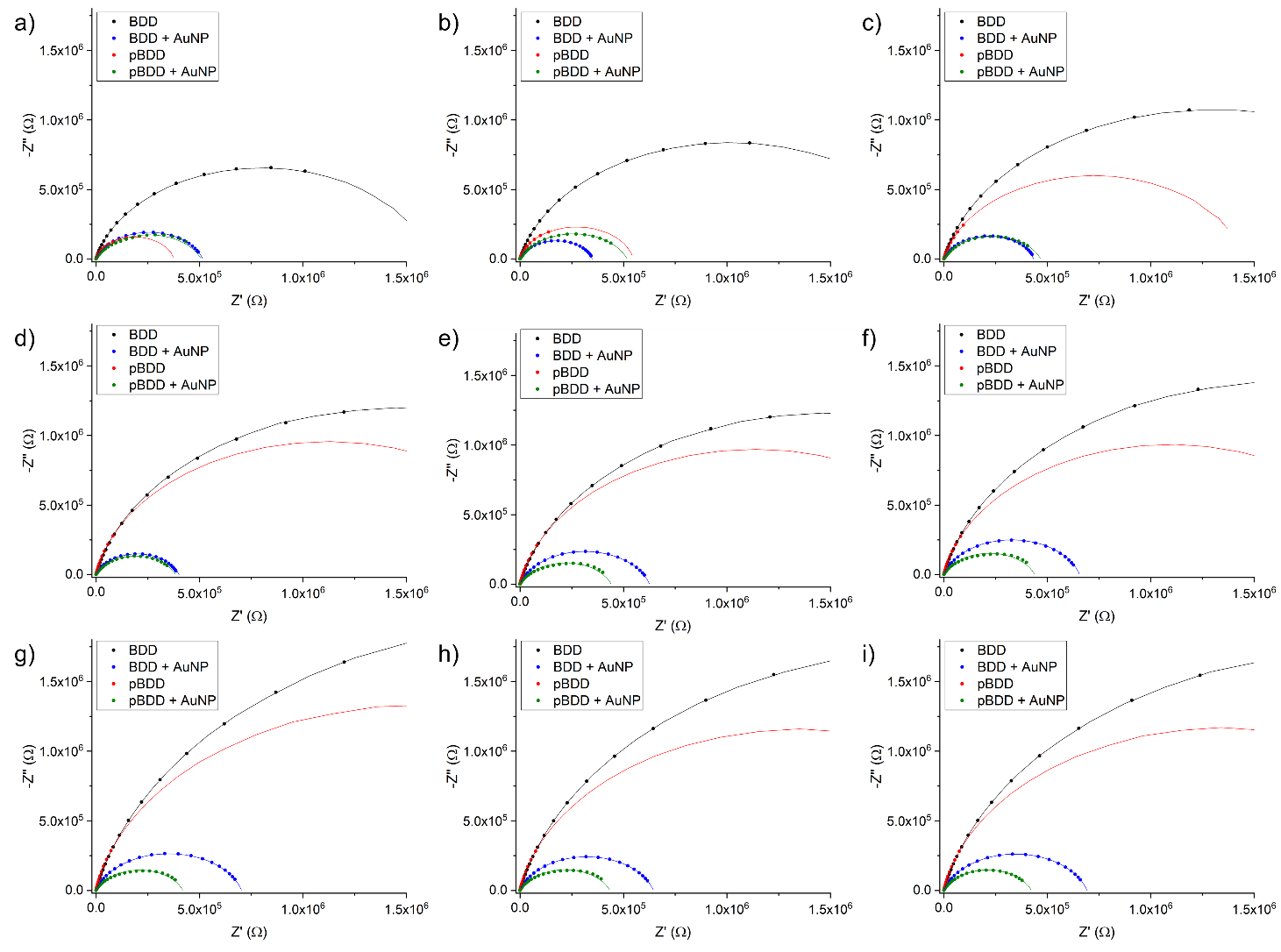
**

Nyquist plot presentation of the EIS data from measurements made over the frequency range 50 kHz – 50 mHz, with 8 points per decade and 10 mV amplitude, for each of the electrodes. The results presented here are from EIS measurements made at a) open circuit potential, 0 M Hg concentration, b) deposition potential (0.35 V), 0 M Hg concentration, c) stripping potential (1.0 V), 0 M Hg concentration, d) open circuit potential, 1 pM Hg concentration, e) deposition potential (0.35 V), 1 pM Hg concentration, f) stripping potential (1.0 V), 1 pM Hg concentration, g) open circuit potential, 1 nM Hg concentration, h) deposition potential (0.35 V), 1 nM Hg concentration and i) stripping potential (1.0 V), 1 nM Hg concentration.

The parameters extracted from the equivalent circuit of the raw EIS data for the BDD and BDD + Au electrodes at the deposition potential (0.35 V).

| **Electrode** | **Hg concentration** | **R_s_ (Ω)** | **R_ct_ (MΩ)** | **C_eff_ (µF/cm^2^)** | **χ^2^** |
| --- | --- | --- | --- | --- | --- |
| BDD | 0 M (control) | 39.8 | 2.04 | 2.14 | 0.06 |
| BDD + AuNP | 0 M (control) | 22.0 | 0.54 | 0.36 | 0.05 |
| BDD | 1 pM | 64.2 | 2.99 | 2.14 | 0.03 |
| BDD + AuNP | 1 pM | 36.3 | 0.52 | 0.36 | 0.08 |
| BDD | 1 nM | 89.3 | 4.23 | 2.21 | 0.02 |
| BDD + AuNP | 1 nM | 103.0 | 0.49 | 0.43 | 0.01 |
| BDD | 1 µM | 99.9 | 4.58 | 2.21 | 0.01 |
| BDD + AuNP | 1 µM | 122.0 | 0.49 | 0.36 | 0.01 |
| BDD | 500 µM | 108.0 | 3.23 | 2.21 | 0.03 |
| BDD + AuNP | 500 µM | 123.0 | 0.58 | 0.36 | 0.07 |
| BDD | 1 mM | 128.0 | 2.28 | 2.00 | 0.10 |
| BDD + AuNP | 1 mM | 127.0 | 0.56 | 0.43 | 0.06 |

The parameters extracted from the equivalent circuit of the raw EIS data for the BDD and BDD + Au electrodes at the stripping potential (1.00 V).

| **Electrode** | **Hg concentration** | **R_s_ (Ω)** | **R_ct_ (MΩ)** | **C_eff_ (µF/cm^2^)** | **χ^2^** |
| --- | --- | --- | --- | --- | --- |
| BDD | 0 M (control) | 42.0 | 2.61 | 2.14 | 0.03 |
| BDD + AuNP | 0 M (control) | 28.5 | 0.42 | 0.36 | 0.07 |
| BDD | 1 pM | 67.6 | 3.38 | 2.21 | 0.02 |
| BDD + AuNP | 1 pM | 46.3 | 0.51 | 0.36 | 0.04 |
| BDD | 1 nM | 90.6 | 4.17 | 2.21 | 0.02 |
| BDD + AuNP | 1 nM | 106.0 | 0.69 | 0.43 | 0.01 |
| BDD | 1 µM | 101.0 | 4.68 | 2.21 | 0.01 |
| BDD + AuNP | 1 µM | 123.0 | 0.72 | 0.43 | 0.01 |
| BDD | 500 µM | 109.0 | 0.81 | 2.14 | 0.04 |
| BDD + AuNP | 500 µM | 126.0 | 0.49 | 0.43 | 0.03 |
| BDD | 1 mM | 132.0 | 2.04 | 2.07 | 0.01 |
| BDD + AuNP | 1 mM | 129.0 | 0.48 | 0.43 | 0.02 |

The parameters extracted from the equivalent circuit fit of the raw EIS data for the pBDD and pBDD + Au electrodes at the deposition potential (0.35 V).

| **Electrode** | **Hg concentration** | **R_1_ (Ω)** | **R_2_ (MΩ)** | **R_3_ (kΩ)** | **C_eff_ (µF/cm^2^)** | **C (µF/cm^2^)** | **χ^2^** |
| --- | --- | --- | --- | --- | --- | --- | --- |
| pBDD | 0 M (control) | 49.0 | 0.55 | 0.02 | 27.5 | 62.79 | 0.04 |
| pBDD + AuNP | 0 M (control) | 34.1 | 0.52 | 2.06 | 0.29 | 15.00 | 0.04 |
| pBDD | 1 pM | 73.6 | 2.27 | 0.03 | 30.21 | 48.50 | 0.09 |
| pBDD + AuNP | 1 pM | 64.5 | 0.44 | 2.37 | 0.64 | 14.00 | 0.03 |
| pBDD | 1 nM | 88.8 | 2.70 | 0.04 | 31.50 | 46.93 | 0.08 |
| pBDD + AuNP | 1 nM | 89.8 | 0.43 | 2.29 | 0.71 | 14.00 | 0.03 |
| pBDD | 1 µM | 108.0 | 3.15 | 0.04 | 32.93 | 46.64 | 0.05 |
| pBDD + AuNP | 1 µM | 107.0 | 0.39 | 2.32 | 0.71 | 16.57 | 0.04 |
| pBDD | 500 µM | 116.0 | 0.52 | 0.03 | 33.14 | 47.07 | 0.08 |
| pBDD + AuNP | 500 µM | 109.0 | 0.27 | 2.33 | 1.07 | 16.64 | 0.08 |
| pBDD | 1 mM | 123.0 | 0.19 | 0.03 | 34.29 | 61.64 | 0.06 |
| pBDD + AuNP | 1 mM | 115.0 | 0.21 | 2.31 | 1.14 | 16.50 | 0.08 |

The parameters extracted from the equivalent circuit fit of the raw EIS data for the pBDD and pBDD + Au electrodes at the stripping potential (1.00 V).

| **Electrode** | **Hg concentration** | **R_1_ (Ω)** | **R_2_ (MΩ)** | **R_3_ (kΩ)** | **C_eff_ (µF/cm^2^)** | **C (µF/cm^2^)** | **χ^2^** |
| --- | --- | --- | --- | --- | --- | --- | --- |
| pBDD | 0 M (control) | 33.3 | 1.45 | 0.02 | 24.21 | 1.21 | 0.02 |
| pBDD + AuNP | 0 M (control) | 38.4 | 0.47 | 2.12 | 0.64 | 14.00 | 0.04 |
| pBDD | 1 pM | 76.0 | 2.19 | 0.04 | 30.71 | 47.71 | 0.09 |
| pBDD + AuNP | 1 pM | 68.0 | 0.44 | 2.35 | 0.64 | 13.50 | 0.03 |
| pBDD | 1 nM | 89.8 | 2.72 | 0.04 | 31.71 | 45.93 | 0.08 |
| pBDD + AuNP | 1 nM | 92.5 | 0.43 | 2.01 | 0.71 | 14.36 | 0.03 |
| pBDD | 1 µM | 109.0 | 2.51 | 0.04 | 33.14 | 48.21 | 0.03 |
| pBDD + AuNP | 1 µM | 110.0 | 0.40 | 1.56 | 0.71 | 15.79 | 0.03 |
| pBDD | 500 µM | 117.0 | 0.63 | 0.04 | 33.07 | 48.14 | 0.07 |
| pBDD + AuNP | 500 µM | 108.0 | 0.34 | 5.87 | 0.79 | 16.93 | 0.08 |
| pBDD | 1 mM | 123.0 | 0.27 | 0.03 | 34.14 | 56.36 | 0.03 |
| pBDD + AuNP | 1 mM | 115.0 | 0.29 | 7.53 | 0.86 | 11.71 | 0.08 |


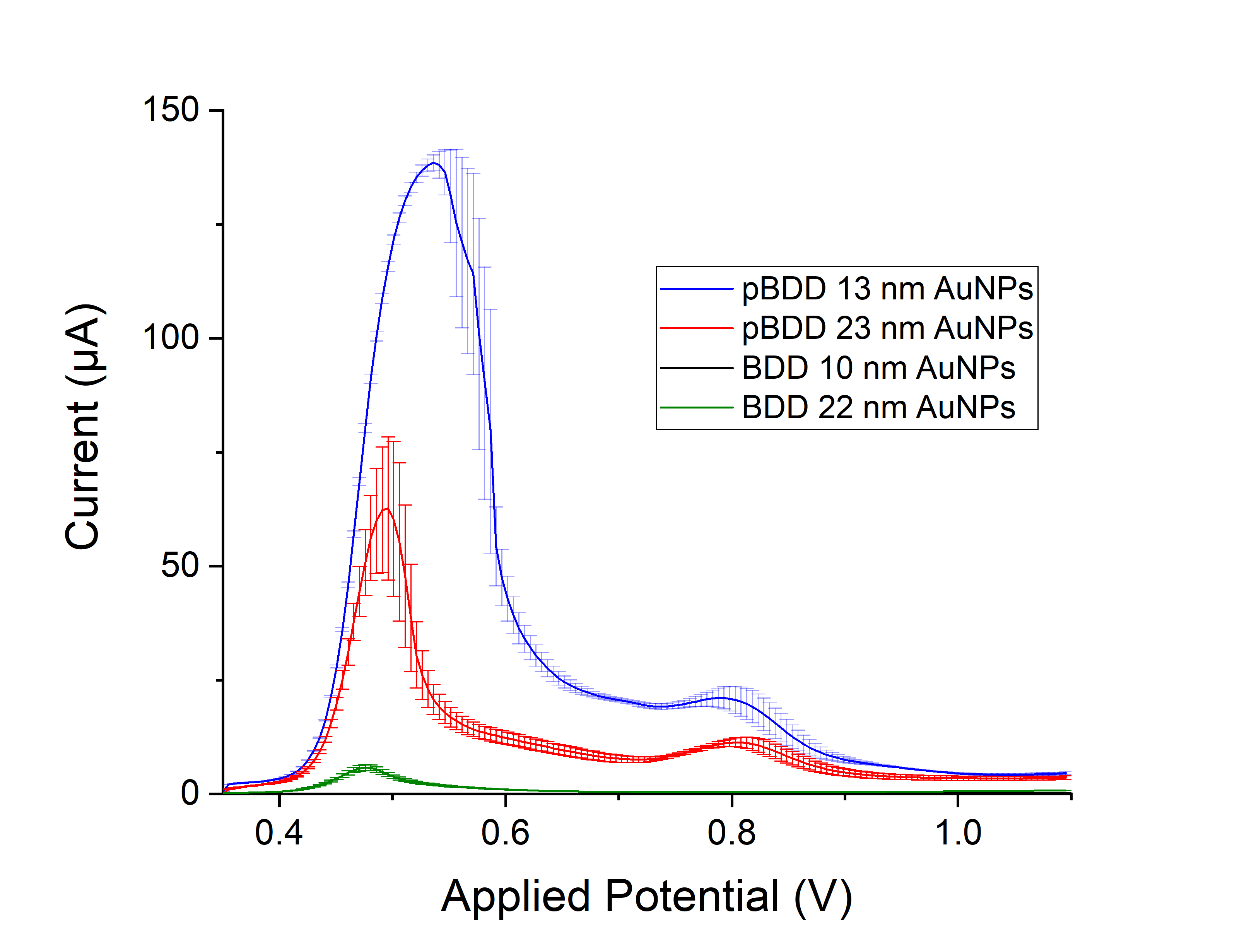


Average SWASV scan and standard error from four repeats of the measurement for mercury detection (mercury concentration 0.1 mM) from the previous paper by the authors (electroanalysis 2019, 31, 1775-1782) showing why 1.0 V was chosen as the stripping potential for EIS measurements, as at this potential there are no peaks on the SWASV plot and so no reaction taking place. All of the mercury has been stripped from the surface of each of the electrodes.
